# Exploring senescence markers as potential drivers of osteoarthritis pain in aging adults^☆^

**DOI:** 10.1016/j.exger.2025.112950

**Published:** 2025-10-30

**Authors:** Muhammad Abbas, Carlos J. Cruz, Javier A. Tamargo, Ania L. Lipat, Rameesha Fareed, Laurel Deaton, Carson Gordon, Alisa Johnson, Blanka Sharma, Christiaan Leeuwenburgh, Kyle D. Allen, Yenisel Cruz-Almeida

**Affiliations:** a Pain Research and Intervention Center of Excellence, University of Florida, Gainesville, FL, United States; b Department of Community Dentistry and Behavioral Science, College of Dentistry, University of Florida, Gainesville, FL, USA; c Institute on Aging, University of Florida, Gainesville, FL, USA; d Department of Applied Physiology and Kinesiology, University of Florida, Gainesville, FL, USA; e Department of Anatomy and Cell Biology, UF Cancer Center, University of Florida, Gainesville, FL, USA; f Pain Research Center, National Center for Complementary & Integrative Health, National Institutes of Health, Bethesda, MD, USA; g J. Crayton Pruitt Family Department of Biomedical Engineering, University of Florida, Gainesville, FL, USA; h Orthopedic and Sports Medicine Institute, University of Florida, Gainesville, FL, USA; i Center for Cognitive Aging and Memory, McKnight Brain Institute, University of Florida, USA; j Department of Neuroscience, College of Medicine, University of Florida, USA

**Keywords:** Osteoarthritis, Chronic pain, Cellular senescence, Senescence-associated secretory phenotype, Inflammation mediators, Senotherapeutics

## Abstract

Senescent cells (SnCs) contribute to various age-related diseases, such as osteoarthritis (OA), a degenerative joint condition that causes persistent pain and reduces physical functioning in older adults. The pathogenesis of OA includes subchondral bone remodeling, synovial inflammation, and cartilage breakdown. Cellular senescence, particularly the pro-inflammatory senescence-associated secretory phenotype (SASP), may have a pivotal role in the progression of OA. SASP factors could exacerbate OA by releasing inflammatory cytokines, chemokines, and proteases, which sensitize nociceptors and accelerate degenerative joint processes, thereby contributing to the chronic pain experienced by OA patients. This contribution of SASPs in chronic pain may lead to mobility limitations and decreased independence of individuals. Thus, the interplay between SASP-driven inflammation and OA pathogenesis may be critical for understanding knee OA pain and functional impairment in older adults. Here, we aim to discuss the parallels between SASP-driven inflammation and OA pathophysiology, which could identify novel therapeutic targets for improving pain management and treatment outcomes in OA.

## 1. Introduction

Osteoarthritis (OA) is a common condition in older age, impacting approximately 7.6 % of the world's population (Collaborators, 2023; Collaborators, 2023) and serving as a leading cause of chronic pain and disability. Despite its high prevalence and burden on older adults, there is currently no Federal Drug Administration (FDA)- or European Medicines Agency (EMA)-approved disease-modifying drug for OA (Katz et al., 2020). While multiple factors — such as genetics, obesity, sex, and joint trauma — influence OA’s development, aging-related cellular and tissue changes are emerging as key contributors to OA pathogenesis (Swain et al., 2020). Cellular senescence is essential to these aging-related processes, a hallmark of biological aging that can drive tissue dysfunction in the OA joint. Senescent cells accumulate in various tissues as people age, driven by factors such as genetic damage, protein stress, metabolic strain, and inflammation (Van Deursen, 2014). These cells release a pro-inflammatory mix of molecules called the senescence-associated secretory phenotype (SASP), identified as factors in age-related tissue damage and illness (Kaur and Farr, 2020). Within the context of OA, SASPs may contribute to cartilage breakdown, synovial inflammation, and subchondral bone remodeling, thereby exacerbating disease progression (Jeon et al., 2018).

Beyond structural damage to the joint, emerging evidence suggests SASPs may act directly on joint nociceptors, fostering peripheral sensitization (Donovan et al., 2025). SASPs associated with cellular senescence, including IL-1, IL-6, and matrix metalloproteinase 3 (MMP-3), can activate nociceptors and sensitize peripheral pain pathways (Jeon et al., 2018). Prolonged and repeated input from the periphery to the central nervous system (i.e., the dorsal horn of the spinal cord) promotes central sensitization that leads to chronic and widespread pain (Donovan et al., 2023). Such pain-related mobility limitations can exacerbate functional decline, disability, and mortality risk (Constantino de Campos et al., 2020; McDonough and Jette, 2010). As a result, clarifying the role of cellular senescence in the pathophysiology of OA and its related pain and disability has the potential to improve interventions to treat this condition.

This review examines the pathophysiological connections between cellular senescence and OA and then considers how these senescence-driven changes might drive pain and functional limitations. Further-more, we address potential therapeutic strategies targeting cellular senescence to mitigate OA progression, with concluding remarks focused on future research directions.

## 2. Pathophysiology of osteoarthritis

OA involves the breakdown of articular cartilage and meniscus, persistent low-grade inflammation (synovitis), synovial fluid breakdown, bone remodeling (e.g., osteophytes and subchondral bone sclerosis), inflammation of the infrapatellar fat pad, and sensitization of joint afferents (Aspden and Saunders, 2019). Although OA can affect various joints, much of the current knowledge derives from research on knee OA, the most common manifestation of OA (Collaborators, 2023). While repeated wear-and-tear with age and trauma over time certainly contribute to the onset of pathophysiologic processes in joint tissue, biological aging of the tissues can promote a pro-inflammatory environment capable of fostering OA (Coppola et al., 2024).

The synovium consists of synovial fibroblasts and is crucial for maintaining joint structure and producing synovial fluid to lubricate the joints. Also found in this tissue are synovial macrophages, which release pro- and anti-inflammatory substances to help stabilize the local microenvironment. Recently, the significance of synovium in the onset and progression of OA has become more widely recognized. Research has increasingly been based on the role of innate immunity, specifically the involvement of resident and circulating macrophages, in synovial inflammation associated with OA (Miller et al., 2020; Van den Bosch et al., 2020; Xie et al., 2019). Additionally, synovitis can associate with OA-related pain (Loeser et al., 2012). As a result, exploring how cellular senescence processes contribute to synovitis in OA will be further explored in the subsequent sections of this review.

Cartilage loss, a hallmark of OA, is driven primarily by ADAMTS enzymes. ADAMTS enzymes selectively cleave aggrecan, a proteoglycan that maintains cartilage’s compressive strength (Fosang and Beier, 2011). The sequential degradation of these extracellular matrix (ECM) components leads to the disintegration of cartilage, impairing the join’s load-bearing and shock absorption capabilities (Shaktivesh et al., 2019). The resultant imbalance between matrix synthesis and degradation accelerates the progressive loss of joint architecture and contributes to the chronicity of OA, where repair mechanisms fail to keep pace with tissue breakdown. This pathogenic cycle underscores the complex interplay between inflammation and tissue degradation, highlighting the multifactorial nature of OA progression.

Mesenchymal stem cells (MSCs) are present in the knee joint and possess self-renewal capabilities. These MSCs can be classified based on their tissue origin into synovial, adipose-derived, and bone marrow-derived types. Among them, the loss and impaired repair of bone marrow MSCs in the subchondral bone contributes to the onset and progression of OA (Camernik et al., 2020). In OA, aging chondrocytes can impair the ability of MSCs to differentiate into chondrocytes (Camernik et al., 2020). Additionally, aging MSCs may lose their ability to regulate the immune response and contribute to OA progression (Camernik et al., 2020). Gao et al. observed that whereas bone marrow MSCs can trigger apoptosis in older chondrocytes, which exacerbates OA, aged chondrocytes can decrease the differentiation and proliferation capacity of these MSCs (Camernik et al., 2020).

## 3. Age-related pain and osteoarthritis

Various age-related mechanisms may contribute to increased pain sensitivity in older adults (Yezierski, 2012). These include age-related anatomical, physiological, and biochemical alterations, loss of homeostatic tissue remodeling, and the somatosensory pathways’ intrinsic plasticity in pain signaling and perception (Yezierski, 2012). Additional factors that may impact increased pain sensitivity with age include disruptions in the hypothalamic-pituitary axis, alteration in autonomic function, and a higher incidence of autoimmune disorders (Yezierski, 2012). Future research in pain and aging should aim to develop clinically relevant animal models and evaluation techniques to better understand the connections between age-related biological changes and variations in pain sensitivity (Yezierski, 2012).

One potential factor contributing to increased pain sensitivity with age is the increase in low-grade systemic inflammation (McGeer et al., 2005; McGeer and McGeer, 2004; Sarkar and Fisher, 2006). This age-related systemic inflammation may predispose older individuals to painful conditions such as osteoarthritis, as chronic inflammation can heighten the sensitivity of peripheral pain receptors, contribute to central sensitization, and activate central stress responses (Chapman et al., 2008; Moalem and Tracey, 2006). The similarities between the biological changes linked with aging and those seen in neuropathic pain suggest that the behavioral effects of aging may be comparable to those following nerve damage (Vierck et al., 2005). Although pre-clinical studies have yielded mixed results, considerable evidence shows that age-related increases in pain sensitivity can occur in various conditions, including regular, inflammatory, and neuropathic states (Vierck et al., 2005). This may be due to a reduced ability of the body’s compensatory mechanisms to manage pain, leading to an environment more prone to pain development (Vierck et al., 2005).

Another factor influencing the shift in sensitivity as people age is the impact of aging on autonomic function. The sympathetic nervous system (SNS) is crucial in maintaining physiological balance under normal conditions and during stress responses. Previous studies indicate that overall SNS activity tends to rise with age. While the exact mechanisms behind this age-related increase in SNS activity remain unclear, there is emerging evidence linking chronic pain to autonomic dysfunction (Yeater et al., 2022). As such, alterations in sympathetic activity may be involved in the initiation and persistence of chronic pain (JW, 1980; Seals and Esler, 2000).

Maladaptive structural changes are associated with nerve remodeling in OA. For example, neurovascular invasion occurs through the osteochondral junction, nerve sprouting occurs in osteophytes, as well as in the synovium and other tissues (Aso et al., 2020; Suri et al., 2007). Joint inflammation is driven by the immune cell infiltration and proinflammatory cytokines secreted by senescent cells in the synovium (i.e. synovitis), and through vascular channels in the osteochondral junction (Jeon et al., 2018; Li et al., 2017; Sellam and Berenbaum, 2010). These mechanisms can contribute to the sensitization of joint afferents which is associated with nociceptive transmission into the dorsal horn of the spinal cord, promoting central sensitization (Eitner et al., 2017; Lluch Girbés et al., 2013). Thus, joint pathology can directly contribute to peripheral and central mechanisms of the joint pain (Syx et al., 2018). OA-related joint inflammation and SASP factors in the cellular microenvironment of joint tissues can therefore have overlapping roles in the pain mechanisms in OA, as will be discussed in the following sections.

### 3.1. Osteoarthritis and age-related mobility disability

Age-related mobility disability may also play a prominent role in the development and progression of OA. The prevalence of mobility disability, which is often defined as difficulty performing a given task (e. g., walking ¼ mile) rises with age, with one epidemiological report finding that mobility disability impacts approximately 30 % of individuals aged 60–69 years, 40 % of individuals aged 70–79 years, and 50 % of individuals aged 80 years or older (Seeman et al., 2010). Mobility disability is also associated with the presence of different health conditions, with OA being one of the leading causes of disability in the world (Vos et al., 2012). Specific measures of physical function, such as gait speed, also demonstrate age-related declines that are exacerbated when OA is present (Duffell et al., 2017; Hollman et al., 2011). The high rates of mobility disability in aging individuals and those with OA is particularly concerning, given the close relationship between mobility, independence, and overall health in older adults (Cawthon et al., 2020; Guralnik et al., 1994; Newman et al., 2006).

From a biological standpoint, age-related mobility declines appear to stem from a complex interaction between a multitude of factors. These factors contribute to biomechanical changes in the affected joints that may promote the progression of OA. For instance, cartilage has been shown to thin with age (Loeser, 2010). Aging also leads to a decline in muscle strength and quality, which may predispose individuals to developing OA (Moore et al., 2014). Indeed, Veronese et al. (2021) found that low skeletal muscle mass at baseline was associated with a greater incidence of symptomatic knee OA after four years (Veronese et al., 2021). Knee extensor muscle weakness has also identified as a risk factor for both radiographic and symptomatic knee OA (Øiestad et al., 2015; Patterson et al., 2023). Behavioral changes, such as slowing gait speeds and changes in joint kinetics and kinematics during movement, also occur with aging (Boyer and Andriacchi, 2016; Hollman et al., 2011). Collectively, these changes can alter joint loading, which can contribute to OA pathology. Joint structure, however, is only one facet of the disease, and is weakly associated with disease symptoms (e.g., pain) (Finan et al., 2013).

When examining the relationship between mobility disability and pain, evidence suggests the opposite relationship is true. Instead of mobility disability preceding pain, studies show that mobility disability becomes more apparent once pain becomes an issue. Indeed, pain has been identified as a risk factor for mobility decline in multiple studies (Buchman et al., 2010; Eggermont et al., 2014; Mottram et al., 2008; Shah et al., 2011). Individuals experiencing pain may also adopt further behavioral compensations to reduce pain, such as adopting a shuffling gait (Duffell et al., 2017; Mills et al., 2013), or reducing physical activity (Wallis et al., 2013). These compensations, however, can further advance OA progression by altering joint loads and by reducing the beneficial anti-inflammatory effects of exercise (Andriacchi and Mündermann, 2006; Brody, 2015). Regardless of the sequential order of these events, the high prevalence and extent of disability associated with mobility-disability in both aging and OA populations is of significant concern.

### 3.2. Cellular senescence and SASP

The accumulation of senescent cells with aging is probably due to several factors, including the decreased capacity of the immune system to remove these cells and their increased resistance to programmed cell death (Wang, 1995). Senescent cells often display a unique gene expression pattern, characterized by increased p16Ink4a and p21Cip1 and heightened anti-apoptotic mechanisms (Kirkland and Tchkonia, 2017). They also typically develop a pro-inflammatory signature called SASP. This secretory profile can include inflammatory chemokines, cytokines, enzymes, growth factors, and interleukins that break down the ECM. These secretions contribute to tissue damage and abnormal remodeling and interfere with normal tissue function (Coppé et al., 2008).

Historically, replicative senescence has been considered the main factor behind the deterioration of tissue maintenance, repair, and regeneration associated with aging. However, further research has revealed that external and internal factors can trigger cellular senescence. These include genomic damage such as telomere shortening, DNA double-strand breaks, epigenetic disruptions, various oncogenic mutations like activated Ras, oxidative stress, and numerous metabolic alterations, especially those affecting mitochondrial function (Booth and Brunet, 2016; Titus et al., 2013; Wiley et al., 2016). Many of these stressors increase the probability of cancer, and senescence-associated growth arrest acts as an effective tumor-suppressing mechanism (Campisi, 2013).

Typically, the senescence response arises from the induction of molecular stress-response pathways. The p53/p21 and p16INK4A/retino-blastoma pathways are vital in driving permanent growth arrest associated with senescence, although other pathways can also contribute to this process (Campisi and d’Adda di Fagagna, 2007). As such, molecular stress-response pathways could be a potential therapeutic target to prevent the pathological effects of cellular senescence on OA.

### 3.3. Chondrocyte senescence in osteoarthritis

The specific joint cells and SASP factors contributing to the OA phenotype are still unclear. Senescence is a complex process characterized by cells’ metabolic, morphological, and physiological changes triggered by several cellular stresses (Coppé et al., 2008; Coryell et al., 2021). It is frequently triggered by growth and arrest signals in cartilage. Senescence markers include the expression of p16INK4A and positive staining for senescence-associated β-galactosidase (Loeser et al., 2016). Measuring the impact of chondrocyte senescence is difficult due to their limited proliferation rate and the overlap between the SASP and OA characteristics (e.g., inflammation). Therefore, understanding the mechanisms behind senescence might lead to therapies that prevent SASP development or selectively remove senescent cells from joint tissues (Loeser et al., 2016).

Aging is associated with a chronic state of low-grade inflammation, also referred to as “inflammaging,” which is recognized as a hallmark of aging (Franceschi et al., 2018; Saavedra et al., 2023). Chronic inflammation is also a key contributor to cellular senescence and OA progression (Han et al., 2024). Inflammaging arises from cumulative activation of the innate immune system, promoting the secretion of pro-inflammatory mediators and SASP factors (Franceschi et al., 2018; Saavedra et al., 2023). In the context of OA, inflammaging may exacerbate chondrocyte dysfunction and matrix breakdown, linking systemic aging processes to local joint pathology (Coskun Benlidayi and Gokce Kutsal, 2022).

**Fig. 1.**
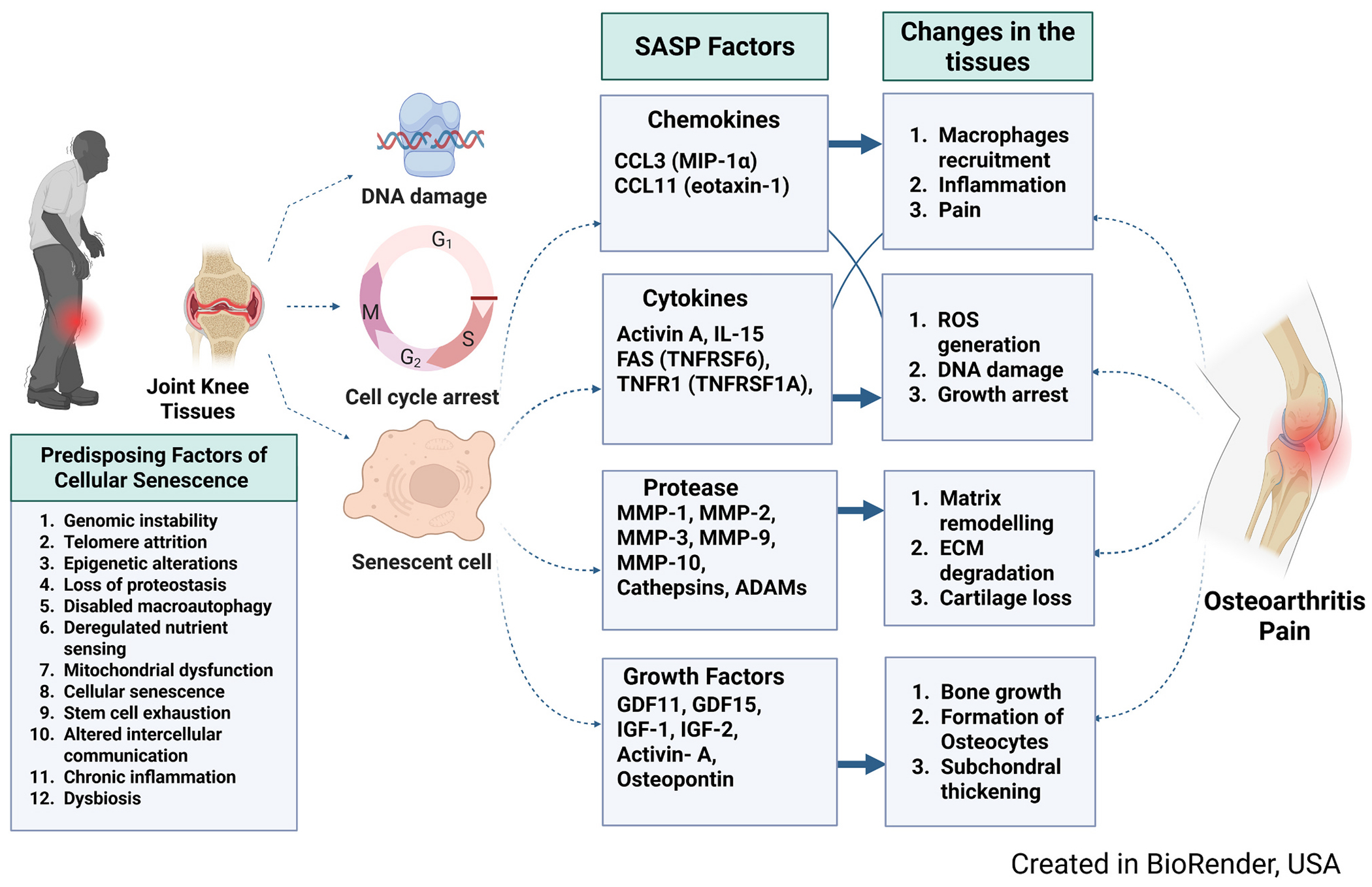
A schematic illustration of predisposing factors of cellular senescence and OA.

Cellular senescence involves several phenotypic changes, as illustrated in Fig. 1. One key mechanism is the induction of senescence by p16, which binds to CDK4 and CDK6, halting the inhibition of retinoblastoma-associated protein (Rb), a cell-cycle repressor protein (Campisi, 2013). Many forms of cellular stress, such as DNA damage from radiation, telomere shortening, ROS, or oncogenic stress, upregulate the expression of P16 (Serrano, 1997), which is strongly associated with aging. Assessing p16 levels can predict an organism’s biological age and has been suggested as a biomarker for cellular senescence (Krishnamurthy et al., 2004). Chondrocytes with high p16 levels also show reduced expression of cartilage-related ECM proteins like aggrecan, while exhibiting an upregulation of ECM-degrading SASP factors such as MMP13 and MMP1. These findings show that chondrocyte senescence is closely associated with age, leading to a metabolic shift that further contributes to cartilage degradation (Coppé et al., 2011). Extracellular vesicles, including exosomes, are nano-sized lipid membrane-bound particles that enable intercellular communication by transporting proteins and RNA between cells (Effenberger et al., 2014; Valadi et al., 2007). Like SASP factors, the secretion of extracellular vesicles increases in these senescent cells (Lehmann et al., 2008) and can lead to premature senescence in nearby cells. One mechanism for this is via the transfer of microRNAs, which further activate downstream senescence-related pathways (Overhoff et al., 2014).

Oxidative stress plays a central role in the initiation and propagation of OA and is a key driver of senescence. Mitochondrial dysfunction, as a result of inflammation, age, or abnormal mechanical loading, leads to elevated levels of reactive oxygen species which overwhelm endogenous antioxidant defenses (Bolduc et al., 2019).Chondrocyte senescence triggered by oxidative stress, occurs mainly by upregulating p53, p21, p38 MAPK, and PI3K/Akt signaling pathways (Dai et al., 2006; Yu and Kim, 2013). This process initiates SASP (Freund et al., 2011). Reactive oxygen species (ROS) can also damage telomeres without cell replication, contributing to senescence, matrix degradation, and chondrocyte apoptosis (Martin et al., 2004). Mitochondrial dysfunction induced by ROS in chondrocytes can enhance inflammatory and matrix-degrading responses to cytokines like IL-1β and TNF through NF-κB activation (Vaamonde-García et al., 2012). Moreover, because chondrocytes respond to mechanical stress, excessive joint loading from acute trauma or instability can increase ROS production and oxidative stress, further promoting senescence in chondrocytes (Harbo et al., 2012; Koike et al., 2015). Senescent cells, in turn, also contribute to ROS production and oxidative stress, resulting in a complex interplay that drives OA progression (von Zglinicki, 2024).

Senescent cells also reside in the bone microenvironment. When assessing the levels of senescence and SASP markers between 6-month and 24-month-old mice, an upregulation of p16 ^^^nk4a, a cell cycle inhibitor, was found in osteoblasts and osteocytes from the trabecular and cortical bone of older mice, indicating the levels of p16^^Ink4a^ increase with age (Farr et al., 2016; McCulloch et al., 2017). There was also a growth in the number of senescent osteocytes in the bone cortex of older animals (McCulloch et al., 2017). Additionally, foci formed because of telomere dysfunction were more common in osteocytes from older mice. These observations indicate that bone loss with age may be partly due to osteocyte senescence, as these cells play a crucial role in bone remodeling (McCulloch et al., 2017).

Another relevant process to cellular senescence is autophagy. This biological process helps cells survive under stress, such as low oxygen or nutrient deprivation, by breaking down and recycling damaged proteins and macromolecules to support protein synthesis (McCulloch et al., 2017). Research on the function of autophagy in cartilage health has been growing, as it may be a key factor connecting aging, cell survival, and OA (McCulloch et al., 2017). Autophagy is predominant in articular cartilage, but this process subsides with age; this reduction is linked to increased chondrocyte death and more significant cartilage damage (Caramés et al., 2015). Additionally, studies have indicated that a chondrocyte-specific deficiency in the autophagy-related protein ATG5 leads to age-related OA hallmarks in mice, along with accelerated chondrocyte apoptosis (Bouderlique et al., 2016; McCulloch et al., 2017). García-Prat et al. explored the link between autophagy and cell survival in muscle stem cells, showing that older satellite cells enter senescence due to reduced autophagy. In developing cells, senescence was triggered by oxidative stress, mitochondrial dysfunction, and disrupted proteostasis (García-Prat et al., 2016). The researchers were able to reverse senescence and restore regenerative abilities by reactivating the autophagy pathway in older cells (García-Prat et al., 2016). Applying this concept to OA, enhancing autophagy in joint tissues could potentially reduce inflammation and promote tissue regeneration, offering a promising therapeutic approach for OA (Ashraf et al., 2017).

### 3.4. Cellular senescence and epigenetic changes in osteoarthritis

OA and cellular senescence involve various epigenetic changes associated with altered cell behavior and disease progression (McCulloch et al., 2017). Several studies have demonstrated the relationship between DNA methylation and cartilage degradation in OA (Hata, 2015). In OA patients, affected chondrocytes show higher levels of DNA methylation in the *Sox9* gene promoter site than normal cartilage, indicating epigenetically impaired chondrogenesis due to decreased *Sox9* expression (Kim et al., 2012). Imagawa et al. reported hypermethylation of CpG sites in the *Col9a1* gene promoter in OA cartilage. Additionally, increased Sox9 binding to the Col9a1 promoter has also been observed in OA chondrocytes (Imagawa et al., 2014). These studies suggest that epigenetic modifications contribute to cartilage degradation and the progression of OA.

Demethylation of CpG sites in the MMP13 promoter increases MMP13 expression, an essential enzyme responsible for cartilage matrix breakdown, in human OA chondrocytes (Bui et al., 2012). Similarly, demethylated promoter regions for other matrix-degrading enzymes, like MMP-3, ADAMTS-4, and MMP-9, have been linked to increased OA expression (Roach et al., 2005). MMP-3, which activates procollagenases, is commonly used to confirm the senescent phenotype in vitro and is considered an essential indicator of senescence. Decreased DNA methylation can lead to p21-dependent cell cycle arrest and senescence in fibroblasts (Young and Smith, 2001).

Moreover, chemically induced DNA demethylation triggers terminal hypertrophic differentiation in chondrocytes, crucial to long bone growth during development, and is reactivated in OA (D’Angelo et al., 2000). While both OA and senescence share enhanced MMP-13 expression, the exact relationship between senescence and cellular hypertrophy, which has been observed in fibroblasts, remains unclear (Demidenko and Blagosklonny, 2008). It’s uncertain whether alterations in DNA methylation directly cause OA or result from the disease. Low DNA methylation levels have been observed at the CpG promoters of SOX9 in human mesenchymal cells, which helps maintain the chondrocyte phenotype, and RUNX2, which promotes chondrocyte hypertrophy—a critical factor in cartilage destruction, leading to mineralization or apoptosis (Ezura et al., 2009). Pro-inflammatory cytokines, including interleukin (IL)-1β, IL-6, and TNF-α, all components of the SASP of senescent cells, are closely linked to OA (Hashimoto et al., 2009). Along with their known role in promoting destructive MMP expression, IL-1β, and TNF-α, along with the IL-6-type cytokine oncostatin M, are known to alter methylation patterns of the IL1B gene, resulting in significant and continued mRNA production (Hashimoto et al., 2009). Understanding the pathways and mechanisms behind these epigenetic modifications and exploring the effects of various epigenetic drugs in OA could open new avenues for therapeutic research and development (McCulloch et al., 2017).

Cellular senescence may have roles beyond inhibiting proliferation, as the SASP involves the synthesis of proinflammatory cytokines and enzymes that degrade the matrix. Notably, SASP regulation appears independent of cell cycle arrest (Loeser et al., 2016). Key SASP factors like IL-1α, IL-6, and CCL2 are present in OA cartilage, making it difficult to determine whether their levels result from senescence or OA (Loeser et al., 2016). Identifying the specific cell type secreting these cytokines is challenging because they are found in synovial fluid and could be produced by other cells, such as those in the meniscus, synovium, or bone (Loeser et al., 2016). This process can also influence neighboring cells by altering paracrine signaling pathways, prompting scientists to explore how senescent cells reshape their microenvironment, which has widespread effects throughout the organism. Like a tumor suppressor, senescence is also characterized by the upregulation of cell cycle inhibitor genes because of oncogenic signals, leading to permanent growth arrest and preventing the uncontrolled proliferation of neoplastic cells (Campisi, 2013). Understanding the pathways and mechanisms behind these epigenetic modifications and exploring the effects of various epigenetic drugs in OA could open new avenues for therapeutic research and development (McCulloch et al., 2017).

### 3.5. SASP and osteoarthritis

Cellular senescence is recognized as a stress response to eliminate damaged cells and promote tissue regeneration (Xie et al., 2021b). However, aging or prolonged stress may make this process incomplete, failing to progress fully through clearance and regeneration. As a result, senescence can contribute to the problem instead of aiding in its resolution (Muñoz-Espín and Serrano, 2014; Xie et al., 2021b). The primary pathological characteristics of OA involve the breakdown of chondrocytes and the degradation of ECM. Chondrocytes, the sole type of cell in articular cartilage, regulate the balance of ECM by producing type II collagen and aggrecan (Glyn-Jones et al., 2015). However, the senescence of various cell types throughout the knee joint contributes to the overall joint degeneration seen in OA (Appleton, 2018; Glyn-Jones et al., 2015). Interestingly, senescent cells are found in the cartilage of individuals with late-stage OA (Xie et al., 2021b).

Articular cartilage is avascular and aneural, primarily composed of a dense ECM rich in type II collagen and aggrecan. It consists mainly of chondrocytes, which maintain the cartilage by balancing ECM production and remodeling through matrix metalloproteinases (MMPs). Early in OA, chondrocytes in the outer layer of cartilage proliferate and form clusters, increasing their activity to repair the tissue. However, as the disease progresses, these chondrocytes lose their role in maintaining cartilage and contribute to matrix degradation and inflammation, mainly through collagen-degrading MMP-13 and aggrecan-degrading ADAMTS-5 (Goldring and Marcu, 2009; Kapoor et al., 2011). This leads to a loss of proteoglycans, erosion of the collagen network, and chondrocyte hypertrophy, resulting in calcified cartilage (Sandell, 2012). Articular chondrocytes typically exhibit minimal proliferation under normal circumstances. Following joint injury, these cells proliferate to aid in cartilage repair and maintain tissue balance. However, chondrocytes that stimulate the multiplication process in this manner are more susceptible to cellular aging, which is crucial for the onset and progression of OA (Appleton, 2018; Ashraf et al., 2017).

Senescent chondrocytes have been observed in cartilage samples from joints affected by OA, especially those removed during total knee arthroplasty (Jeon et al., 2017). These cells accumulate with age and are found in higher numbers in OA cartilage than in healthy cartilage from similarly aged individuals (Philipot et al., 2014). Their presence near OA lesions, rather than in unaffected areas, suggests a link to the disease (Price et al., 2002). Since chondrocytes rarely replicate under normal conditions, the presence of senescent chondrocytes in OA joints may be due to senescence mechanisms other than replicative senescence. Regardless, these senescent cells secrete factors associated with senescence, including inflammatory mediators and ECM-degrading enzymes (Mobasheri, 2013).

SnCs trigger an innate immune response that helps remove them (Krizhanovsky et al., 2008). Natural killer (NK) cells detect distressed cells through specific surface receptors and ligands. Some SnCs express ligands that bind to NK cell receptors, such as NK group 2D (NKG2D) and nonclassical MHC I molecules like HLA-E (Coppé et al., 2010; Sagiv et al., 2016). Macrophages are crucial in clearing senescent cells from tissues and stimulating repair and regeneration (Wynn et al., 2013; Yun et al., 2015). Senescent stromal cells can attract or induce a specific subset of macrophages that temporarily display certain senescence markers, implying that the cells showing markers like p16INK4A and senescence-associated β-galactosidase (SA β-gal) in OA-affected tissues may be macrophages (Hall et al., 2017). These macrophage types, stimulated by IL-4, could have regenerative effects (Dray and Read, 2007).

### 3.6. Age-related pain and SASP

Joint pain is a primary reason for patients seeking medical intervention. Although pain does not always directly correlate with the level of tissue damage in the joint, it remains a key aspect of OA. The exact biological mechanisms behind joint pain are not fully understood, but local inflammation is considered a contributing factor (Malfait and Schnitzer, 2013). Increased levels of TNF-α, a factor associated with the SASP, are connected to pain in OA joints (Donovan et al., 2024). Studies linking the SnCs and pain include observations that pain relief often occurs after removing SnCs, even before any noticeable changes in tissue structure occur (Jeon et al., 2017).

**Fig. 2.**
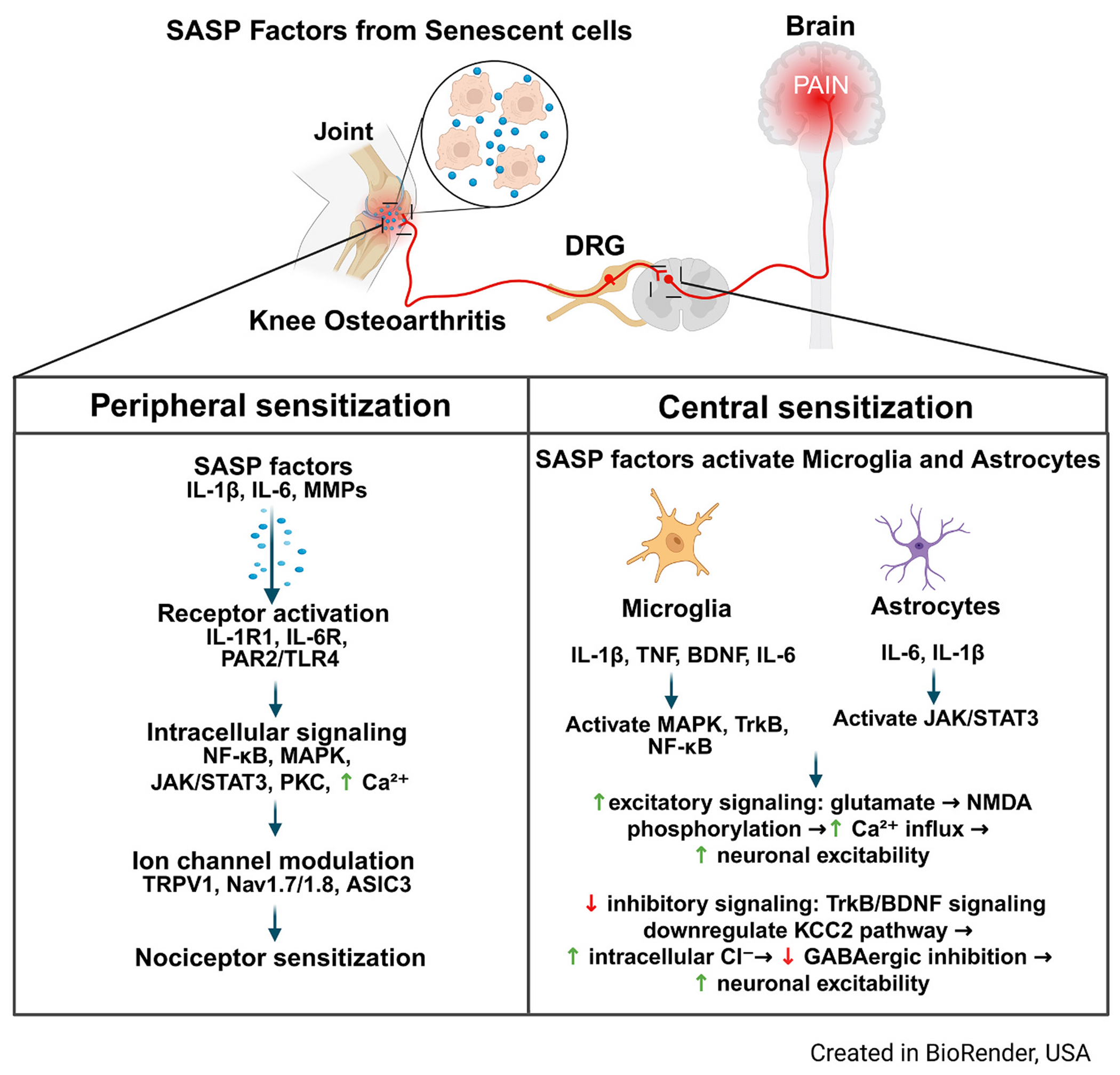
Molecular mechanisms of nociceptive pain mediated by proinflammatory SASP factors in knee OA.

Interestingly, primary sensory neurons in the dorsal root ganglia (DRG) have been shown to have upregulated markers of senescence (P21 and P16) with aging, along with SASP factor IL6, which may promote the sensitization of peripheral nociceptors (Donovan et al., 2025). The upregulation of Trpv1^+^ nociceptors that co-express both p21 and IL6, as observed with increasing age and post-injury, indicates that this specific population can be affected by such cytokine synthesis. This population of nociceptors (Trpv1^+^) is previously known to be highly involved in pain sensation (Donovan et al., 2025). Clinical studies emphasize the role of aberrant DRG excitability in pain as temporary effectiveness was seen in chronic pain patients as a result of peripheral anesthetics (Richards and McMahon, 2013) or nerve blockage (Hayek and Shah, 2014). Moreover, there was an increase in transcriptomic signatures of senescence across multiple organ systems (i.e., SenMayo signature) in human DRG in pain states which hints towards the impact of senescence in the DRG on pain human pain outcomes (Donovan et al., 2025). As shown in Fig. 2, SASP factors (IL-1b, IL-6, MMPs) bind to receptors on joint afferents (IL-1R1, IL-6R, PAR2/TLR4), influencing intracellular signaling (e.g., NF-kB) and ultimately contributing to peripheral sensitization (Lee et al., 2013). These peripheral nervous system changes also influence central mechanisms involving microglia and astrocytes, which secrete SASP factors and can alter excitatory signaling mechanisms (e.g., Ca^2+^ influx) (Lee et al., 2011).

The sensitization of peripheral nociceptors and pathophysiologic processes driven by cellular senescence at the joint level (e.g., cartilage loss, synovitis) is a potential source of OA-related pain that needs further exploration. The synovium is a probable source of pain-inducing cytokines, though other cell types may also contribute (Loeser et al., 2012; Miller et al., 2014). In various joint tissues, immune cells can also release cytokines that contribute to pain.

SASP factors secreted by SnCs and cytokines released by immune cells can contribute to pain, potentially affecting OA symptoms. For instance, inflammatory mediators such as TNF-α, and arachidonatederived PGE2/COX-2, interleukins (IL-1 and IL-6) are known to exacerbate pain (Dray and Read, 2007). PGE2 is a significant factor in heightened pain sensations through its interaction with E prostanoid receptors (EP1–4) on peripheral sensory neurons, which relay pain signals to the brain (Dray and Read, 2007). In human OA tissues, elevated levels of PGE2 and COX-2 have been observed in the synovium, meniscus, and bone (Attur et al., 2008; Sato et al., 2011). Identifying SASP factors responsible for pain, whether produced by immune cells, synovial cells, or other joint tissue cells. This will enhance our understanding of OA symptoms and the potential effectiveness of senolytic therapies (Orita et al., 2011).

### 3.7. Age-related mobility-disability and SASP

Emerging evidence in animal and human studies suggests that SASP factors also play a role in age-related mobility-disability. In mice, transplanting senescent cells into young and old mice led to persistent physical dysfunction and reduced survival (Xu et al., 2018). In humans, higher concentrations of activin A, ICAM1, MMP7, VEGFA, eotaxin, and IL6 were associated with worse lower extremity physical function and handgrip strength (Fielding et al., 2022). Several senescence biomarkers, such as vascular endothelial growth factor A (VEGFA), tumor necrosis factor receptor 1 (TNFR1), and matrix metallopeptidase 7 (MMP7), have been associated with the onset of significant and persistent mobility disability (Fielding et al., 2024). Moreover, in older, obese women, higher levels of p16INK4a + in thigh adipose tissue were associated with worse grip strength, walking abilities, and self-perceived mobility (Justice et al., 2018). The findings from these studies suggest that cellular senescence in OA may contribute to impaired physical function, which is often observed in those suffering from the condition.

The limited number of studies investigating the relationship between senescence markers and physical function in individuals with knee OA have primarily focused on the associations between serum levels of proinflammatory cytokines and self-reported physical function. Two studies found that self-reported physical function was not associated with IL-6. IL-6, IL-8, IL-10, or TNF-α concentrations (Imamura et al., 2015; Santos et al., 2011). Ruan et al. (2019), similarly found a significant association between IL-8 serum concentrations and self-reported physical function in their study. Barker et al. (2019), found significant associations between physical function and IL-1β, IL-4, IL-5, IL-12, IL-13, and interferon (IFN)-γ concentrations. Given the limited number of studies investigating this relationship in humans, further investigation of the role of SASP factors in mobility disability in OA is warranted.

### 3.8. Potential therapeutic approaches for osteoarthritis-related pain

Existing treatments for OA include NSAIDs for pain relief, physiotherapy, intra-articular hyaluronate injections (viscosupplementation), and platelet-rich plasma injections that provide short-term relief. However, these options cannot prevent or reverse the progression of the disease. Recent breakthroughs in understanding the underlying mechanisms of OA may offer new treatment approaches beyond simply managing symptoms (Xie et al., 2021a). Studies on cellular senescence’s role in OA development have introduced promising treatment strategies, such as removing senescent cells, inhibiting the SASP, and rejuvenating stem cells (Xie et al., 2021a). To decrease the buildup of senescent cells, researchers are exploring senotherapeutics, an emerging class of drugs (Ansari et al., 2024). Senolytics and senomorphics are two therapeutics that have shown the potential to reduce aging-related diseases in mouse models and are now tested in human trials (Kang, 2019).

Senolytics, which include plant-based polyphenols, small molecules, and anti-apoptotic inhibitors, have shown potential in preclinical studies for removing senescent cells from joint tissues (Ansari et al., 2024). Senolytics help reduce the effects of age-related conditions, such as OA, by counteracting the role of SASP in disease progression. Senescent cells have anti-apoptotic pathways that protect them from undergoing programmed cell death, making them resilient to various forms of stress. Senolytic agents specifically target the critical proteins involved in these pathways, promoting significant changes that enable the targeted elimination of SnCs (Ansari et al., 2024). These drugs target aging cells by either removing them (senolytics) or blocking their harmful effects (senomorphics) and are seen as promising methods to counteract human senescence (Kaur and Farr, 2020). Senolytics selectively cause apoptosis in SnCs, while senomorphics focus on inhibiting SASP factors associated with pro-inflammatory signaling and tissue injury (Kang, 2019). Several senolytics are under investigation in rodents’ studies and human clinical trials for OA, such as UBX0101, a compound that inhibits the binding of p53 to MDM2, an E3 ubiquitin ligase responsible for p53 degradation. In a mouse model of post-traumatic OA, local intra-articular injection of UBX0101 selectively eliminated the SnCs, reduced proteoglycan loss, and improved OA-related outcomes such as pain and articular cartilage breakdown (Jeon et al., 2017). However, in a randomized clinical trial, UBX0101 did not improve painful symptoms in individuals with OA, highlighting the need to potentially recalibrate drug-delivery parameters (e.g., formulation, frequency) (Lane et al., 2021). Moreover, Seno-lytics often triggers apoptosis, specifically in SnCs, by blocking survival pathways active in SnCs but inactive in healthy cells. For instance, administering navitoclax (ABT-263), a dual inhibitor of BCL-2 and BCL-XL, to irradiated or naturally aged mice caused the elimination of senescent hematopoietic stem cells in the bone marrow and senescent muscle stem cells, ultimately promoting cellular rejuvenation (Coryell et al., 2021). The combination of dasatinib and quercetin potently clears SnCs (Coryell et al., 2021). In aged mice, treatment with dasatinib and quercetin lowered the presence of senescent osteocytes in bone, reduced osteoclast formation and bone degradation, improved bone mineral absorption and thickness, and significantly enhanced the structure of both trabecular and cortical bone (Coryell et al., 2021). While the effects of several senotherapeutics in rodents are promising, more work is needed to properly identify viable candidates that will successfully translate for OA treatment. Specifically, challenges in translation (e.g., UBX0101) highlight the importance of including a wide range of behavioral pain assessments, such as both stimulus- (e.g., von Frey) and non-stimulus-evoked (e.g., gait assessment) assessments, as well as evaluating the relative effect size of any noted improvements that could correspond with a clinically meaningful reduction in pain (Deuis et al., 2017). Equally important is the selection of the OA model. While no preclinical model perfectly represents human OA conditions, assessing senotherapeutics across multiple models (e.g., ACLT, MCLT+MMT, spontaneous OA) may help ensure that a consistent effect is observed (Teeple et al., 2013). Finally, future studies could consider incorporating overlapping factors between OA, aging, and cellular senescence — such as comorbidities (e.g., obesity) (Palmer et al., 2022) which may more accurately reflect the health conditions of the target population for these senotherapeutics.

Targeting inflammation-related pathways and molecules is a well-established therapeutic approach. Numerous senomorphic compounds have been identified to suppress SASP-related pathways without trig-gering cell death. These contain inhibitors of IκB kinase and NFκB such as NEMO-binding domain peptides (Tilstra et al., 2012), JAK inhibitors (like ruxolitinib) (Xu et al., 2015), ATM inhibitors (such as KU-60019) (Kang et al., 2017), compounds that block progerin-lamin A/C binding like JH4 (Lee et al., 2016), activators of PDGF and fibroblast growth factor signaling (Bae et al., 2016), and inhibitors of TGFβ receptor type 2 and p21 such as Mmu-miR-291a-3p (Bae et al., 2016). Since SASP expression is linked to OA-related pain, targeting these factors presents a promising treatment strategy, though selecting the appropriate target is crucial for effectiveness and precision.

## 4. Conclusion and future directions

Senescent cells and their associated SASP factors influence OA progression by promoting inflammation, impairing tissue repair, and accelerating joint damage. These processes exacerbate pain, decreasing mobility and physical function, particularly in older adults. Future studies should focus on identifying tissue-specific SASP factors to better understand the role of senescent cells in different joint structures in OA. Additionally, validating specific senescence markers, such as p16^^INK4a^, p21, and β-galactosidase, could enhance OA diagnosis, monitor disease progression, and evaluate the efficacy of senotherapeutics. Recently, senolytics have been shown to alleviate pain-like behaviors in aged mice with OA, offering promise as a potential disease-modifying therapy for OA. However, further work is needed to develop targeted therapies that will successfully translate from preclinical models to humans by halting or reversing the pathways of OA, pain, and physical impairment. It also remains unclear whether targeting senescence locally in the joint will be sufficient to treat OA in humans, or if systemic therapies will be required. Additionally, because epigenetic modification — unlike genetic changes — are reversible and associated with both OA and cellular senescence, targeting them may offer new therapeutic opportunities to slow or manage OA pathogenesis.

## Data Availability

No data was used for the research described in the article.
